# Proline confers acid stress tolerance to *Bacillus megaterium* G18

**DOI:** 10.1038/s41598-022-12709-0

**Published:** 2022-05-25

**Authors:** Gunajit Goswami, Dibya Jyoti Hazarika, Naimisha Chowdhury, Sudipta Sankar Bora, Unmona Sarmah, Romen Singh Naorem, Robin Chandra Boro, Madhumita Barooah

**Affiliations:** 1 DBT-North East Centre for Agricultural Biotechnology, Assam Agricultural University, Jorhat, Assam 785013 India; 2 Department of Agricultural Biotechnology, Assam Agricultural University, Jorhat, Assam 785013 India

**Keywords:** Molecular biology, Microbiology, Bacteria, Bacteriology

## Abstract

Proline plays a multifunctional role in several organisms including bacteria in conferring protection under stress conditions. In this paper we report the role of proline in conferring acid tolerance to *Bacillus megaterium* G18. An acid susceptible mutant of *B. megaterium* G18 which required proline for its growth under acid stress condition was generated through Tn5 mutagenesis. Further, targeted inactivation of *proC* involved in osmo-adaptive proline synthesis in *B. megaterium* G18 resulted in the loss of ability of the bacterium to grow at low pH (pH 4.5). Exogenous supply of proline (1 mM) to the growth medium restored the ability of the mutant cells to grow at pH 4.5 which was not the same in case of other osmoprotectants tested. Proline was produced and secreted to extracellular medium by *B. megaterium* G18 when growing in low pH condition as evidenced by the use of *Escherichia coli* proline auxotrophs and HPLC analysis. Further, pHT01 vector based expression of full length *proC* gene in the ∆proC mutant cells restored the survival capacity of the mutant cells in acidic pH, suggesting that proline production is an important strategy employed by *B. megaterium* G18 to survive under acid stress induced osmotic stress.

## Introduction

The multifarious role of proline in several different organisms including plants and animals is well established. Proline provides protective benefits against abiotic and biotic stresses in a broad range of organisms^1–6^. Involvement of proline biosynthetic machineries during osmoprotection has been well described. It is a known osmoprotectant providing protection under drought and salt stress by stabilizing proteins, subcellular structure and cell membrane^7,8^; Proline acts as a thermoprotectant to prevent protein aggregation during heat stress in *Escherichia coli*^9^. The imino acid is reported to protect the cellular functions by scavenging Reactive Oxygen Species (ROS)^10^. Proline is an important source of carbon and nitrogen for several bacteria including *E. coli*, *Pseudomonas putida*, *Bradyrhizobium japonicum* and *Helicobacter pylori* growing under various nutrient conditions^11–15^. The oxidative stress alleviating property of this amino acid has been explored in eukaryotes such as fungi, plants, and animals^2,3,16,17^. It is understood that proline provides protection against oxidative stress by maintaining intracellular redox homeostasis and increasing catalase activity^16,18^. Despite the number of reported functions of proline, its role in conferring tolerance to acidic stress in bacteria has not been investigated in detail and very few reports describe its direct role in the mechanism of acid stress protection.

In *Bacillus subtilis*, proline required for osmotic adaptation is synthesized by the enzymes encoded by the proJ-proA*-proH genes, whereas the production of anabolic proline is catalyzed by enzymes encoded by *proB-proA-proI* genes^19,20^. The disruption of the proJ-proA*-proH cassette resulted in osmo-sensitive phenotype in *B. subtilis*^21^. Similar genetic machineries operate in the production of osmo-adaptive proline and proline required for anabolic process in *B. megaterium*^22^. Both sets of genes encode the identical three enzymes, viz., γ-glutamate-5-kinase (by *proB, proJ*), γ-glutamyl phosphate reductase, also known as glutamate-5-semialdehyde dehydrogenase *(*by proA, proA*) and Δ^1^-pyrroline-5-carboxylate reductase (by *proI*, *proH*)^22^. However, the genes associated with proline synthesis are annotated as *proB-proA*-*proC* in the published sequenced genome of *B. megaterium* ATCC14581 (GenBank: CP035094.1; BioProject: PRJNA514939) and accordingly mentioned in the present study*.*

Previously, we had reported isolation of an acid tolerant isolate of *B. megaterium* with plant growth promoting (PGP) activity and ability to tolerate low pH (4.5) condition. *B. megaterium,* recently shifted to a new genus called *Pristia* and currently named as *Pristia megaterium*^23^ is a big rod-shaped Gram-positive soil bacterium with long history of industrial use^24,25^. Besides industrial application, *B. megaterium* also has agricultural significance as different strains of this bacterium possess plant growth promoting (PGP) traits^26–29^. Our earlier studies indicate that low pH tolerance (4.5) in *B. megaterium* is mediated by an array of mechanisms^30,31^. Among the several mechanisms adopted by *B. megaterium* to adapt to acid stress, proline appeared to be important as the genes (*proA*, *proB* and *proC*) involved in proline biosynthesis showed increased expression along with high intracellular proline accumulation during acid stress^31^. Taking cue from results of our previous investigation, we delved further to understand the role of proline in conferring acid tolerance in *B. megaterium* G18 and report the results of our investigation in this paper.

## Results

### Transposon mutagenesis and isolation of mutant

The Tn5 was introduced into *B. megaterium* G18 using the suicide plasmid pSUP5011^32^. Transconjugants were selected on NA plates containing neomycin followed by heat treatment. Over one hundred Nm^R^ colonies were obtained after incubation, of which several colonies were randomly selected for further studies.

### Confirmation of the Tn5 mutants

There were no morphological differences between the transconjugant and the wild-type bacterium. Among the 100 transconjugants screened for their acid susceptible characteristics, 5 transconjugants were unable to survive at pH 4.5 and thus were selected for further studies. The presence of Tn5 was confirmed in the acid susceptible transconjugants. The transposon Tn5 harbors the neomycin phosphotransferase II (*nptII*) gene and upon successful integration of the Tn5 element into the host chromosome, the transconjugant displays neomycin resistance. Therefore successful integration of Tn5 element into the host chromosome can be confirmed by amplifying the *nptII* gene from the host genomic DNA. Among the several neomycin resistant transconjugates, amplification of the characteristic 1.5 kb product of *nptII* gene in 5 transconjugants indicated the successful integration of the Tn5 into the genome of *B. megaterium* and the acid susceptible character was due to Tn5 integration. The agarose gel electrophoresis micrograph showing the *nptII* amplification is depicted in Supplementary file, Fig. S2. Among the *nptII* positive transconsjugates, BA5 was selected for further analysis.

### Proline requirement test of BA5

**Figure 1:**
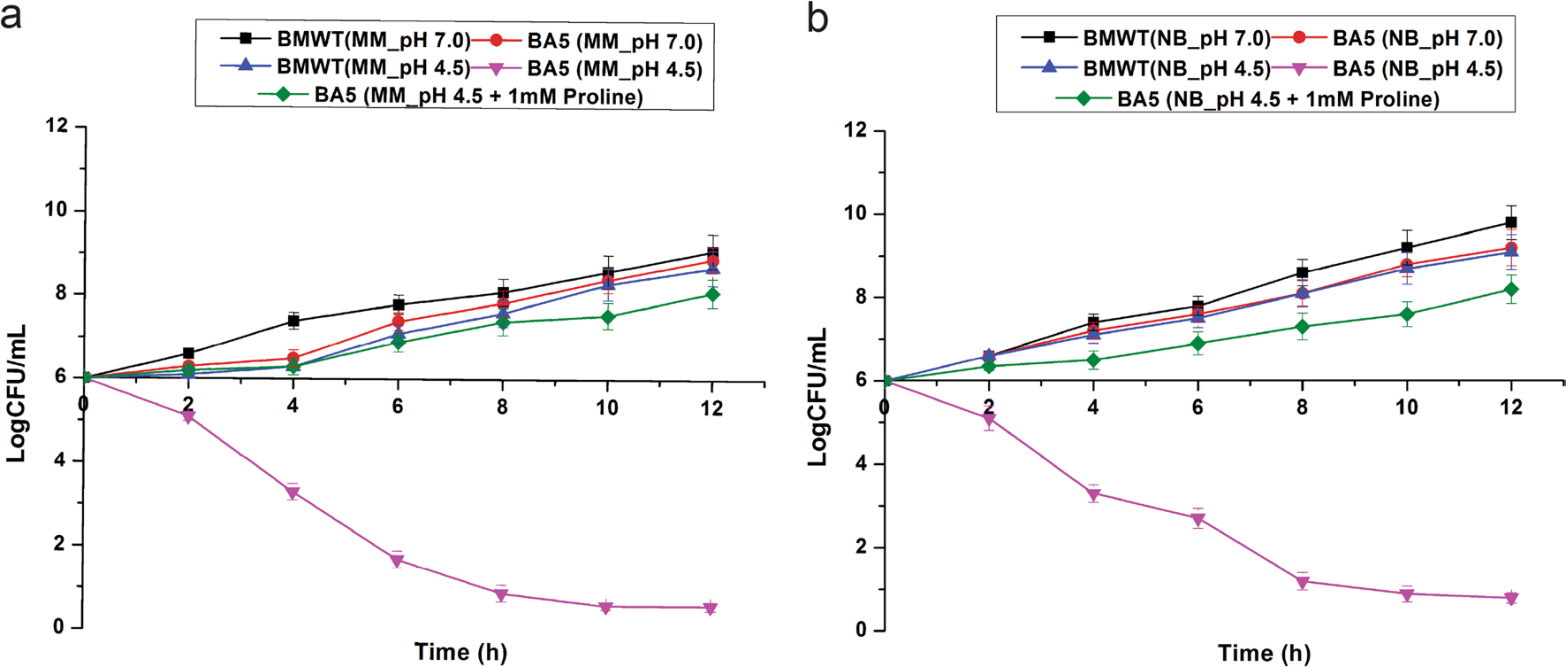
Acid tolerance and proline requirement test of the Tn5 mutant (BA5). (**a**) The growth of the Tn5 mutant (BA5) in minimal media (MM) adjusted to pH 4.5 was tested in presence and absence of l-proline and compared with the growth of WT at the same stressed media (**b**) The growth of the Tn5 mutant (BA5) in Nutrient Broth (NB) adjusted to pH 4.5 was tested in presence and absence of l-proline and compared with the growth of WT at the same stressed media. The error bar represents standard error of mean (n = 3).

The mutant, tested for requirement of proline for growth in NB and Minimal media at low pH revealed inability of the mutant cells to grow in acidic conditions (pH 4.5) in the absence of l-proline. The mutant cells were however, able to grow in the same NB and Minimal media with pH adjusted to neutral conditions (pH 7.0) even in the absence of l-proline. Supplementation of the media with l-proline (1 mM) restored growth of the mutant at pH 4.5 indicating the requirement for proline to grow in acidic condition (Fig. 1).

### Targeted disruption *proC* gene in *B. megaterium* G18

The plasmid pMUTproCi was transformed into the host *B. megaterium* G18 and selected on NA plate containing erythromycin (0.4 µg/mL). The erythromycin resistant colonies appearing with a transformation frequency of (4 × 10^–3^ transformants/ng pMUTproCi) were considered as putative pMUTproCi transformants. The mutants were confirmed based on their cultural characteristics as well as insertion of *ermAM* gene through PCR amplification*.* The pMUTIN4 vector integrates into the host chromosome by a single recombination event between the cloned locus (*proC*i) and the corresponding chromosomal locus. Since the vector harbors the *ermAM* gene, successful integration into the host chromosome results in conferring erythromycin resistance to the mutant cells which were selected based on their growth in erythromycin containing medium (Supplementary file, Fig. S3).

### Acid and salt tolerance of the *proC* mutant (BM13A)

**Figure 2:**
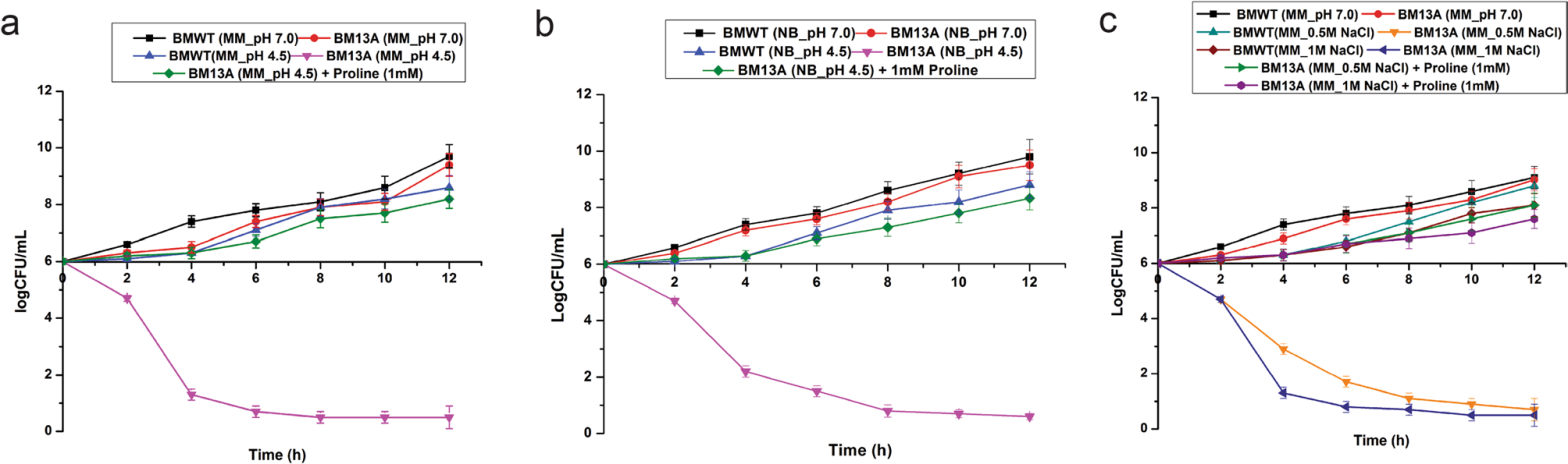
Acid and salt tolerance response of the *proC* mutant (BM13A). (**a**) Comparing the growth of the *proC* mutant (BM13A) in minimal media (MM) adjusted to pH 4.5 in presence and absence of l-proline and compared with its growth at pH 7.0 and also with the growth of the WT at pH 7.0 and pH 4.5 at the same media. (**b**) Comparing the growth of the *proC* mutant (BM13A) in Nutrient Broth (NB) adjusted to pH 4.5 in presence and absence of l-proline and compared with its growth at pH 7.0 and also with the growth of the WT at pH 7.0 and pH 4.5 at the same media. (**c**) Comparing the growth of the proC mutant (BM13A) in minimal media (MM) of pH 7.0 in presence and absence of 0.5 M and 1 M NaCl, comparing its growth in the same media with the WT as well as the effect of l-proline in alleviating the salt stress of BM13A. The error bar represents standard error of mean (n = 3).

The *proC* mutant (BM13A) was tested for its acid susceptible character in both NB and minimal media (pH 4.5) with and without the addition of l-proline. The experiment revealed reduced survival of the mutant as observed from the reduction in colony forming unit (from 6 logCFU/mL at 0 h to 0.5–1 logCFU/mL after 12 h) when grown in absence of proline. However, supplementation of the culture medium with l-proline (1 mM) enabled the mutant cells to survive and grow to attain 8 logCFU/mL after 12 h (Fig. 2A,B). The mutant cells were also had poor growth (from initial 6 log CFU/mL to ~ 0.8–1 logCFU/mL after 12 h) under salt stress condition (minimal medium containing 500 mM and 1 M NaCl); Similar to the acidic stress condition, supplemention with 1 mM l-proline revived the growth of the mutants in salt stress (7–8 logCFU/mL after 12 h) (Fig. 2C). The wild type cells had normal growth (8 logCFU/mL after 12 h) both in acid and salt stressed conditions.

### Effect of osmoprotectants on the growth of the mutants at low pH

**Figure 3:**
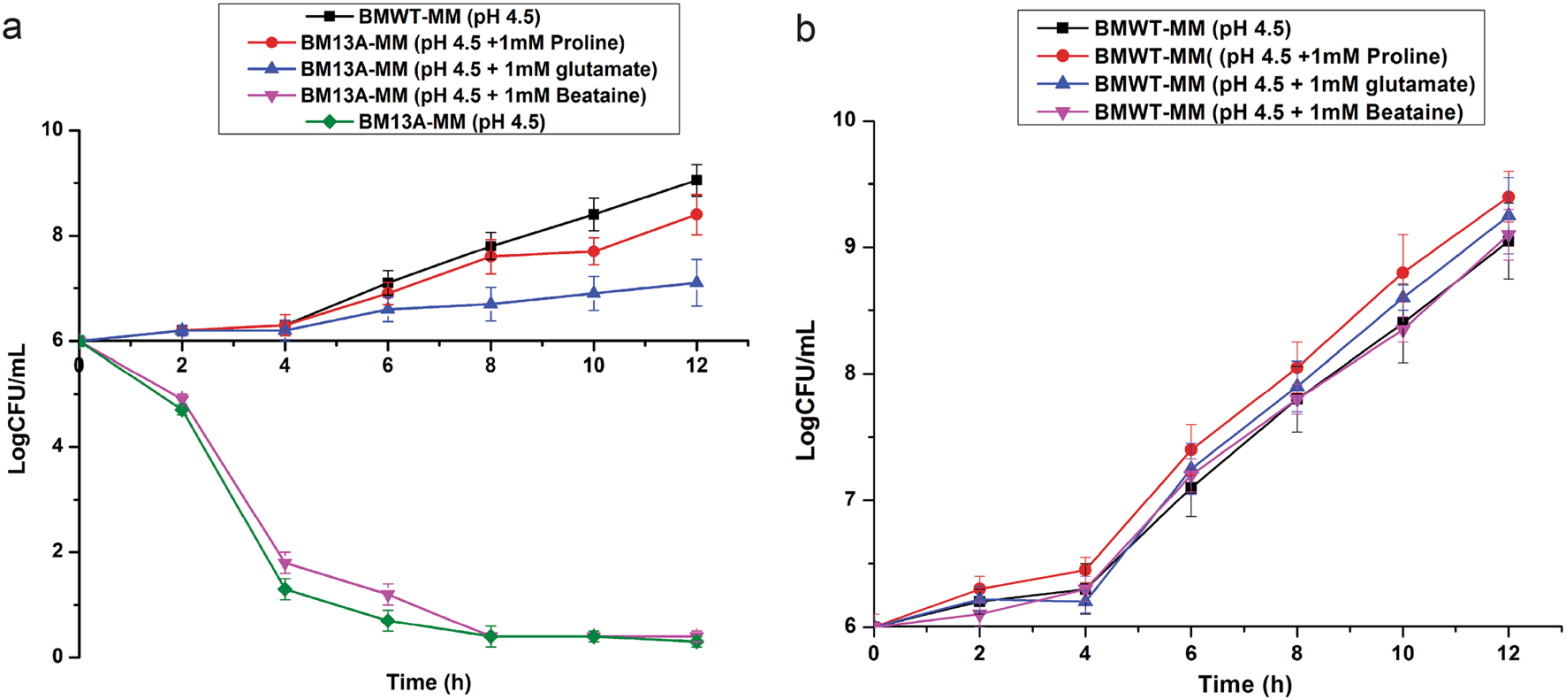
(**a**) Effect of osmoprotectants on the growth of the *proC* mutant (BM13A) in acid stress condition (pH 4.5). The growth of the *proC* mutant (BM13A) was compared by growing it in Minimal Media (MM) with and without the addition of osmoprotectants separately viz*.*, l-proline, l-glutamate and beataine and also compared its growth with the WT grown in MM adjusted to pH 4.5. The error bar represents standard error of mean (n = 3). (**b**) Effect of osmoprotectants on the growth of *B. megaterium* G18 in acid stress condition. The growth of the *proC* mutant (BM13A) was compared by growing it in Minimal Media (MM) with and without the addition of osmoprotectants separately viz*.*, l-proline, l-glutamate and beataine.

Proline is a reported osmolyte associated with maintaining osmotic balance in *B. megaterium* cells^22^ and it was expected that mutation in the gene related to proline biosynthesis may affect the growth of the *proC* mutant (BM13A). Therefore, besides l-proline, other osmoprotectant viz. betaine and l-glutamate were substituted to test if they had similar effect on the growth of the mutant and the WT cells. Both l-proline and l-glutamate improved the growth of the mutant; however, l-glutamate (initial 6 logCFU/mL increased to 7 logCFU/mL after 12 h) could not improve the growth in the mutant cells to the extent provided by l-proline (initial 6 logCFU/mL increased to 8.1 logCFU/mL after 12 h). The growth rate of the WT cells at pH 4.5 also improved on supplementation of l-proline and l-glutamate compared to the growth at pH 4.5 without proline and glutamate. Other osmoprotectants tested did not appear to have any effect on the growth of the WT. This indicated that under acid stress, proline as an osmoprotectant provides better protection and improves survivability in *B. megaterium* G18 under acid stress condition compared to other osmoprotectants (Fig. 3).

### Production of extracellular proline by *B. megaterium* G18 under acid stress

**Figure 4:**
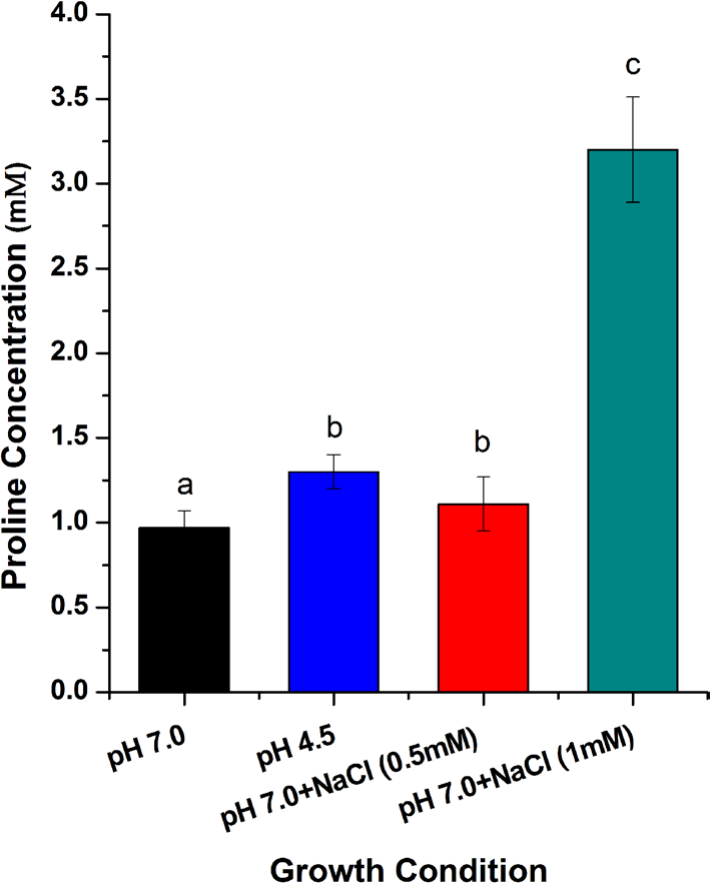
Spectrophotometric estimation of extracellular proline production by *B. megaterium G18* after growing in minimal media adjusted to pH 4.5 for 6 h. The error bar represents standard error of mean (n = 3).

**Figure 5:**
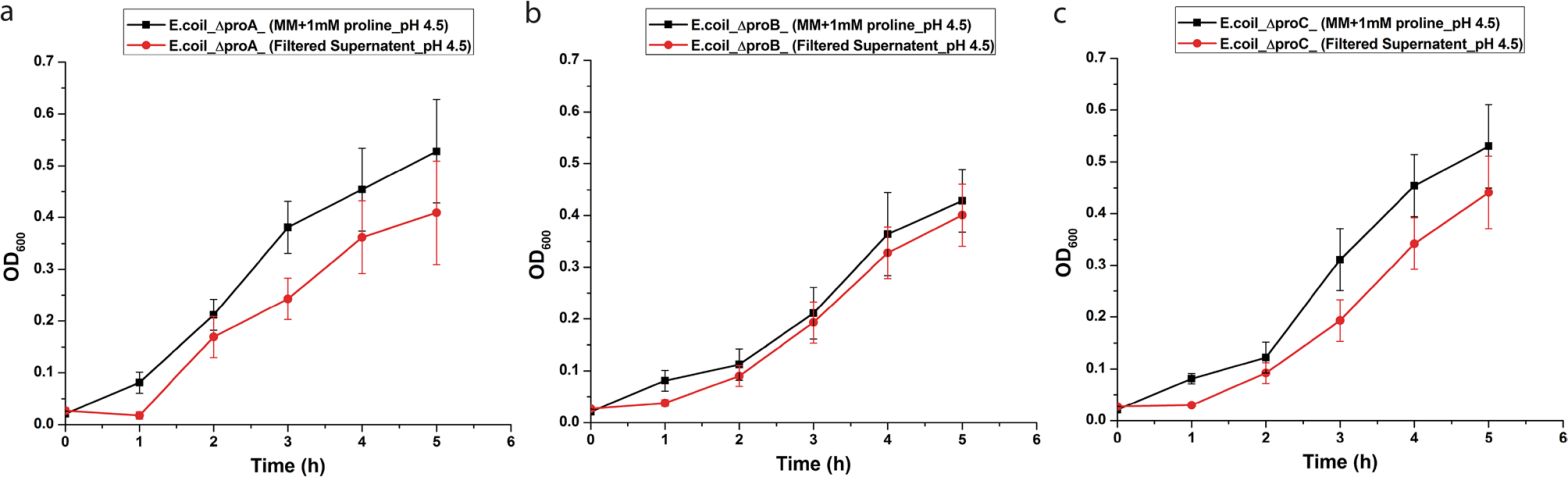
Validation of proline released by *B. megaterium* G18 in the culture media under acid stress using *E. coli* proline auxotrophs. (**a**) Growth of *E. coli* ΔproA strain (**b**) Growth of *E. coli* ΔproB strain and (**c**) Growth of *E. coli* ΔproC strain in the filtered media supernatant in which *B. megaterium* G18 was grown for 6 h; growth of the each strain in MM supplemented with l-proline serves as control. The error bar represents standard error of mean (n = 3).

**Figure 6:**
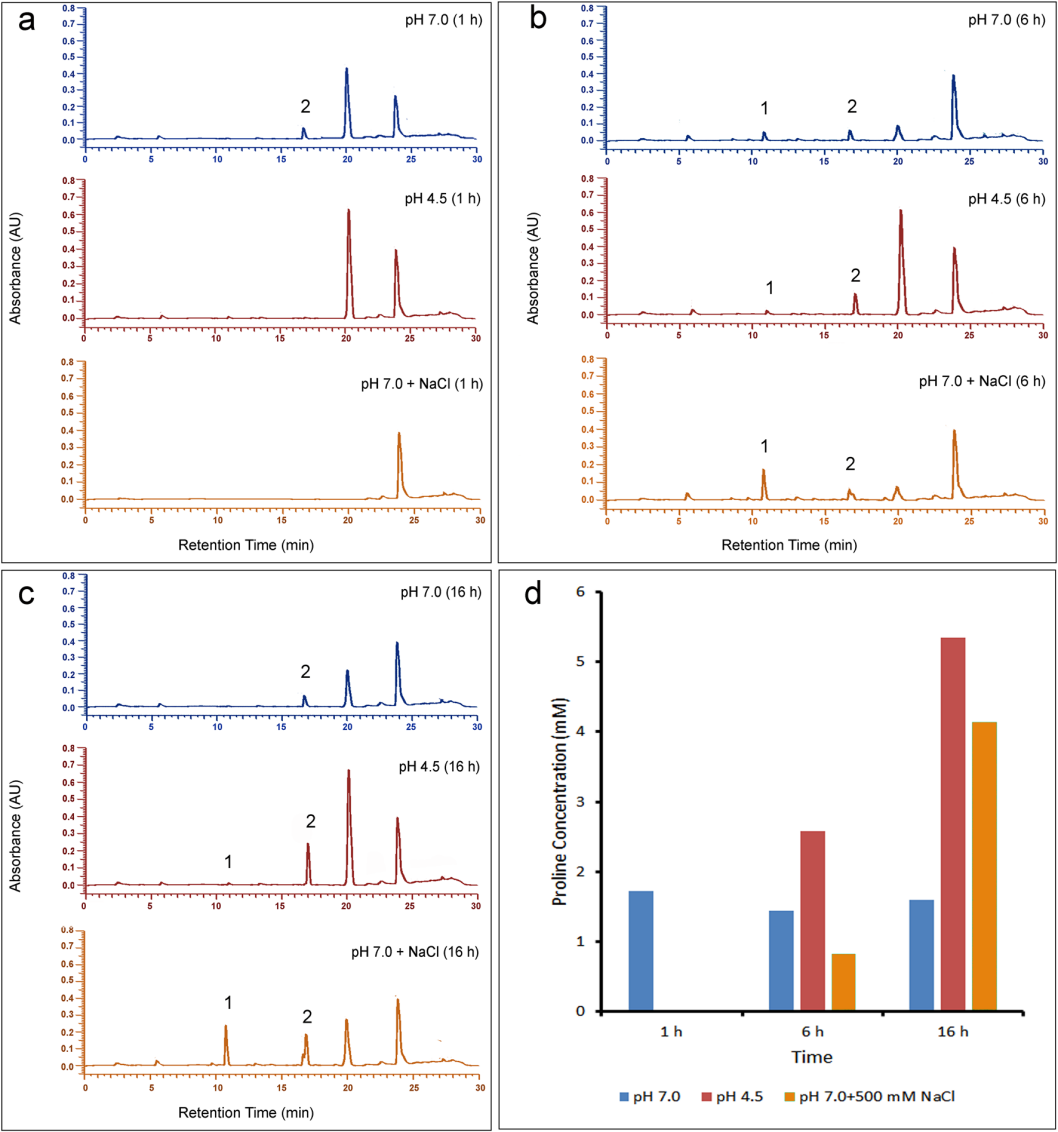
Detection of extracellular of proline secreted under acid stress by *B. megaterium* G18 using HPLC. The isolate was grown in minimal media (MM) adjusted to pH 7.0 and pH 4.5 as well as MM of pH 7.0 containing 0.5 M NaCl for 1 h, 6 h and 16 h. Determination of l-proline in the media supernatant of all the 3 conditions (**a**) after 1 h (**b**) after 6 h and (**c**) after 16 h, the numerical 1 and 2 in figure (**a**–**c**) represents the peak of l-glutamate and l-proline respectively. (**d**) Graphical representation of the concentration of proline produced in each time point.

**Figure 7:**
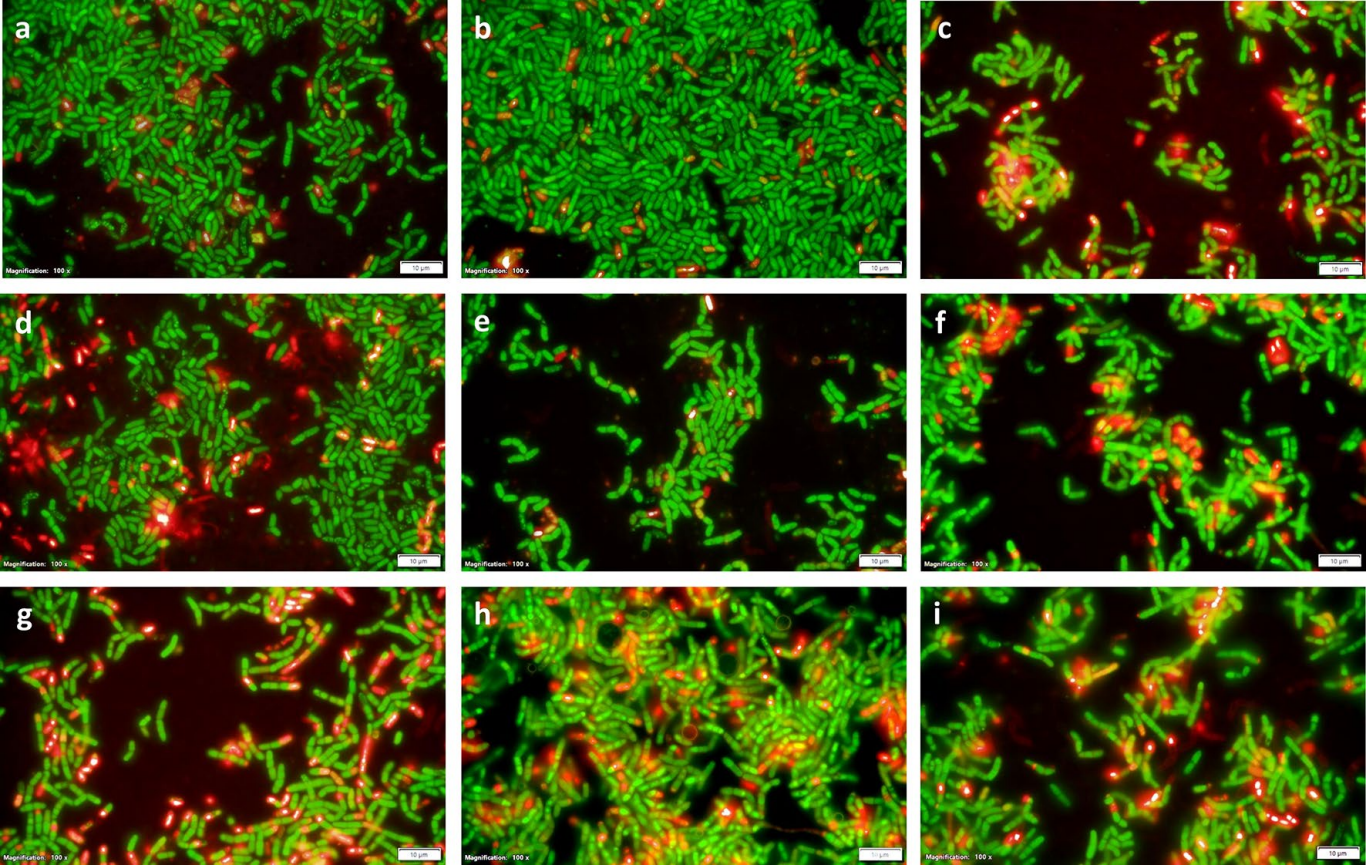
Representative results of Live/Dead BacLight staining on *B. megaterium* G18 cells grown in Minimal Media of pH 7.0, pH 4.5 and Minimal Media of pH 7.0 containing 0.5 M NaCl for 1 h, 6 h and 16 h. The cells were incubated for 5 min in the presence of 1 μL of 1.67 mM propidium iodide (PI) and 1.67 mM SYTO 9. Image of bacterial cells at 500 nm (green) for SYTO9 signal and at 635 nm (red) for PI signal were superimposed using Image J tool. (**a**–**c**) Images of *B. megaterium* G18 cells grown in Minimal media of pH 7.0 for 1 h, 6 h, and 16 h respectively. (**d**–**f**) Images of *B. megaterium* G18 cells grown in Minimal media of pH 4.5 for 1 h, 6 h and 16 h respectively. (**g**–**i**) Images of *B. megaterium* G18 cells grown in Minimal media of pH 7.0 amended with 0.5 M NaCl for 1 h, 6 h and 16 h respectively (magnification: ×100 with oil immersion for all images).

The culture supernatant of *B. megaterium* G18 grown at pH 4.5 accumulated proline to a concentration of 1.3 mM while, the culture supernatant of *B. megaterium* G18 grown at pH 7.0 had accumulation of only 0.97 mM proline after 6 h of incubation (Fig. 4). The bacterial cultures grown for 1 h did not accumulate proline under all the conditions tested. The extracellular secretion of proline by *B. megaterium* G18 in the culture media was further supported by the ability of *E. coli* proline auxotrophs to grow in the filtered supernatant of *B. megaterium* G18 cultures (Fig. 5). The HPLC analysis of the media supernatant of 6 h and 16 h old culture *B. megaterium* G18 revealed proline concentration of 2.5 mM and 5 mM respectively lending further, credence to the extracellular secretion of proline by *B. megaterium* G18 under acid stress (Fig. 6). We also performed a fluorescent microscopic analysis (Fig. 7) to ascertain if the proline detected in the growth media was due to rupture of the cells. The microscopic study revealed a small fraction of cells as either dead or with leaky membrane as some of the cells did uptake the propidium iodide dye. Although, the contribution of the dead cells on the extracellular proline concentration cannot be denied as evident from the uptake of propidium iodide stain which can only penetrate dead cells or cells with leaky membrane, its effect will be very marginal given that only a minuscule cell populations were dead or with leaky membrane as evident from the microscopic images (Fig. 7).

### qRT-PCR analysis

**Figure 8:**
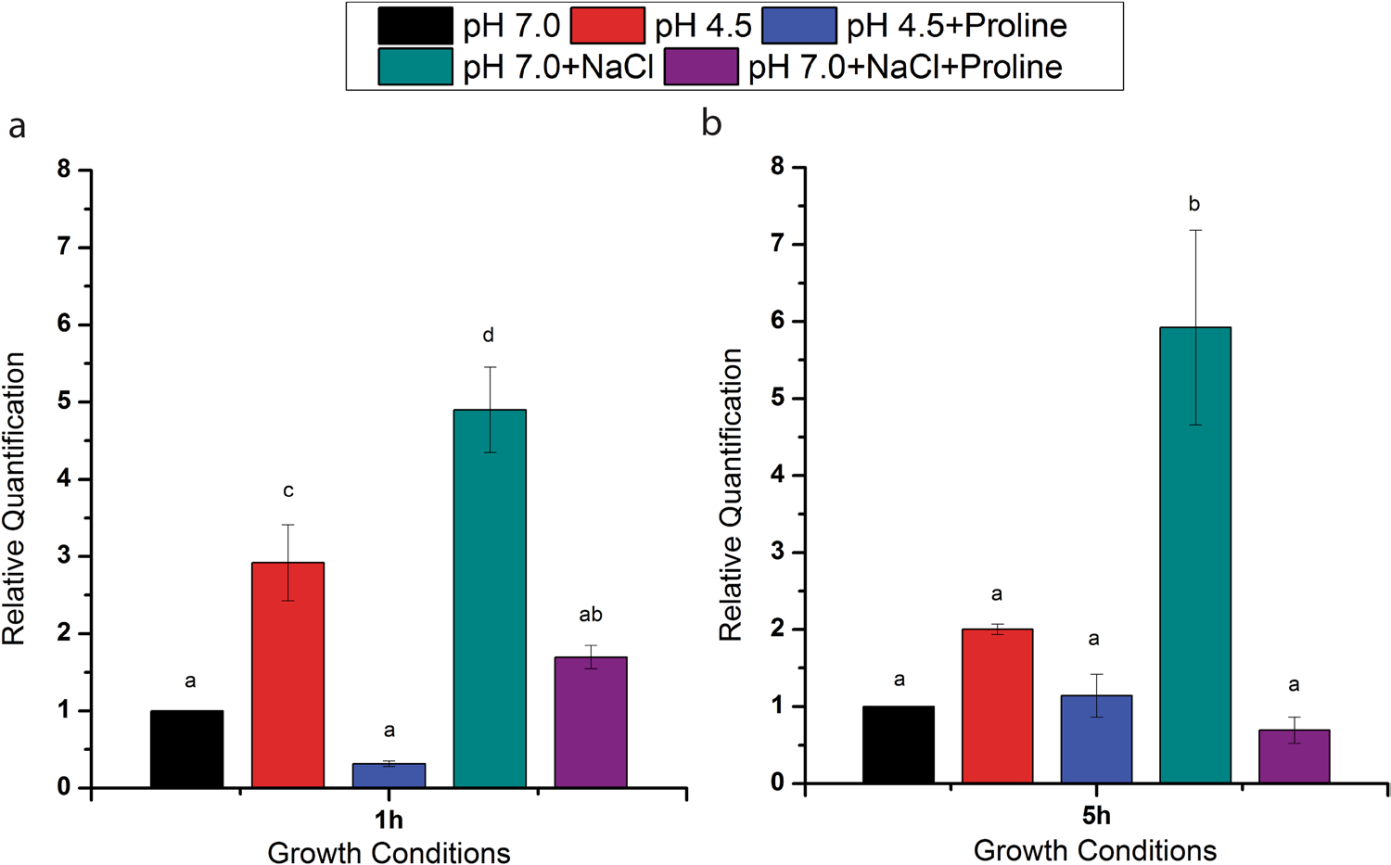
Comparing the relative expression of *proC* in acid- (pH 4.5) and salt stressed *B. megaterium* G18 cells with the non-stressed *B. megaterium* G18 cells grown at 1 h and 5 h. The error bar represents standard error of mean (n = 3). Different letter(s) above the indicates significant difference (p ≤ 0.05).

The qPCR analysis revealed increased expression of *proC* in the cells grown at low pH (4.5) and salt stress condition compared with the cells grown under neutral pH conditions at both the time points (1 h and 5 h). Further, in the presence of proline in low pH (4.5) medium, the bacterium did not show any significant difference in the expression of *proC* when compared to the expression in the cells cultivated in unstressed condition (Fig. 8).

### Expression of *proC* gene in acid susceptible mutant

To validate the involvement of Δ^1^-pyrroline-5-carboxylate reductase in acid stress, the full length *proC* gene was expressed in acid susceptible *B. megaterium* ΔproC cells (Supplementary file, Fig. S4). Quantitative real-time PCR analysis showed upregulation of *proC* transcript in *B. megaterium* pHT01-proC^+^ cells by RQ values of 1.2 and 3.1 at 1 h and 5 h of exposure to acid stress, respectively, compared to the wild type cells grown at pH 7.0 (Supplementary file, Fig. S5). Wild type cells exposed to acid stress also followed the similar pattern compared to that grown in pH 7.0, however the expression level was more in wild type compared to the *B. megaterium* pHT01-proC^+^ cells. Expression of *proC* gene using pHT01 expression vector revealed that *proC* gene expression restored the survival of the susceptible mutant cells. The mutant cells after *proC* expression showed similar growth behavior as the wild type cells grown at pH 4.5 (Supplementary file, Fig. S6).

### Microscopic analysis to understand the effect acid and salt stress on *B. megaterium* G18 and BM13A

**Figure 9:**
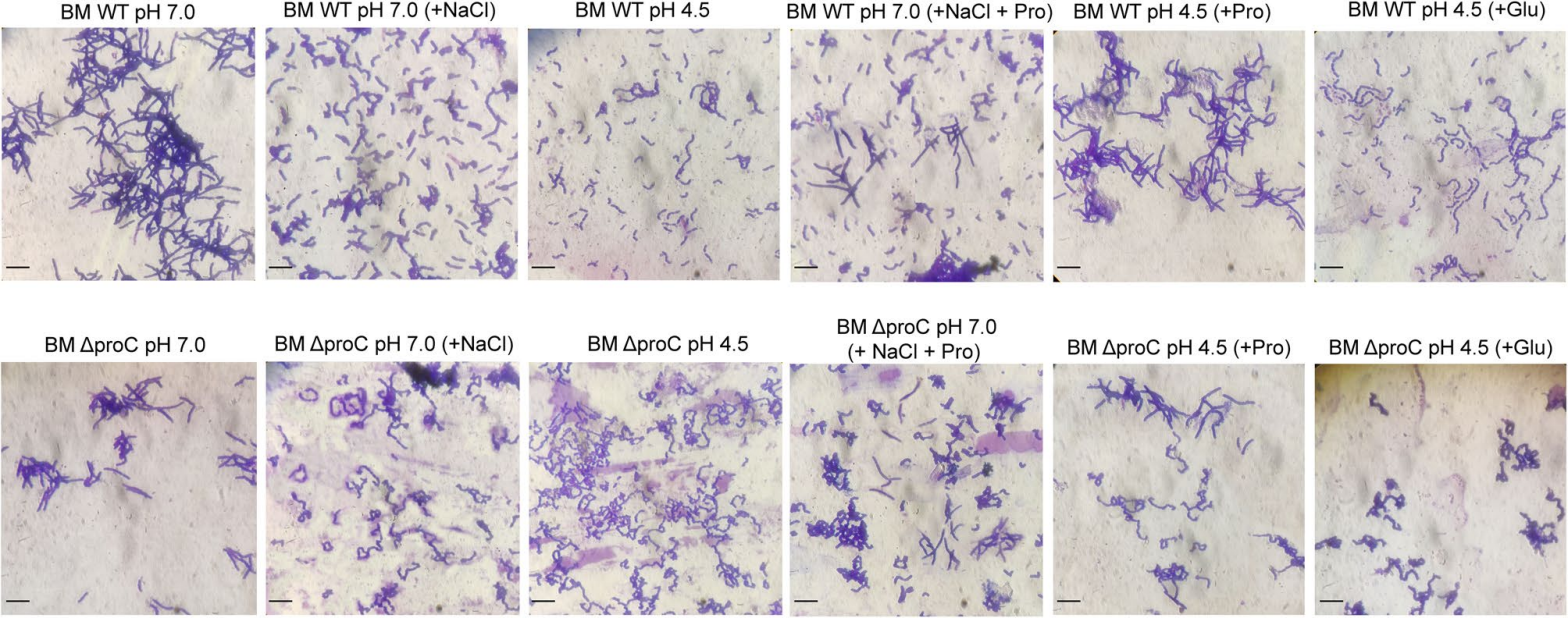
Microscopic analyses to examine the effect acid (pH 4.5) and salt stress (NaCl 0.5 M) on *B. megaterium* G18 and BM13A morphology in presence of l-proline (Pro) and l-glutamate (Glu). The *B. megaterium* G18 and BM13A cells (6 logCFU/mL) were grown in minimal media in acid and salt stressed condition for 1 h, harvested by centrifugation at 6000 rpm for 5 min, gram stained and observed under light microscope.

Microscopic analysis revealed that acid stress had impact on the cell morphology and was similar to the morphology induced by salt stress; however, the effect on the cellular morphology was more pronounced in the cells of the mutant BM13A grown under acid and salt stress of cells compared to the wild type cells. Addition of l-proline to the media restored the cell morphology to the similar state of the cells grown in MM of pH 7.0. The WT cells regained normal cell morphology more rapidly upon proline supplementation compared to the BM13A cells. Glutamate did not have any significant effect on the restoration of cellular morphology in both wild type and mutant cells (Fig. 9).

## Discussion

The role of proline in conferring protection against acid stress (pH 4.5) in *B. megaterium* G18 was investigated by analyzing the acid stress response of wild-type and *proC* mutant of *B. megaterium* G18. Our earlier study had revealed an increased expression of genes encoding the enzymes involved in proline biosynthesis in *B. megaterium* G18 when exposed to low pH (4.5) condition^31^. To gain further insight into the role of proline in providing acid tolerance, we adopted a transposon mediated mutagenesis approach to generate an acid susceptible mutant of *B. megaterium* G18 (BA5) that could not grow at low pH without proline supplementation. However, it was not clear whether the acid susceptible character was due to mutation in the genes involved in proline biosynthesis, as transposon mediated mutants are reported to display polar effect^33,34^. We therefore, targeted *proC*- the final gene encoding for Δ^1-^pyrroline-5-carboxylate reductase enzyme of the proline biosynthetic pathway. In *B. megaterium* proline involved with conferring omso-adaptation is produced through a pathway involving at least 3 genes^22^. Given the increased expression of the *proC* gene (RQ = 18.5 ± 1.4) compared to the *proA* (RQ = 8.2 ± 0.5) and *proB* (RQ = 8.5 ± 0.6) as revealed from our earlier study^31^, we chose to inactivate the last gene (*proC*) of the proline biosynthetic pathway to determine the effect of proline in conferring acid tolerance. The generated *proC* mutant (BM13A) had much reduced growth at low pH (pH 4.5), showed improved growth at pH 4.5 when the medium was supplemented with 1 mM l-proline. Our study provides strong evidence that proline confers protection and increases the ability of *B. megaterium* G18 to survive in acid stress condition. Proline is a multifunctional amino acid associated with several important functions including carbon and nitrogen metabolism^35^, protein synthesis, and protection against various environmental factors such as drought^36^, metal toxicity^16,37^, osmotic stress^22,38,39^, ultraviolent irradiation^40^, unfolded protein stress^6,9^, and oxidative stress^2,6,16,17^. Although, it is evident that proline provides acid tolerance in *B. megaterium* G18, the actual mechanism of its action warrants thorough investigation. Several studies have reported that proline is mainly associated with conferring osmotic stress^19,21,41^, assuming that the low pH condition induces the bacterial cells also to experience osmotic stress, we further performed experiment where the WT and the mutant cells were grown in presence of other osmoprotectants to see if they were equally effective in restoring the growth of the bacterial cells. Our study suggested that proline is the most preferred solute followed by glutamate under acid stress among the all osmoprotectants tested. However, *B. megaterium* does not possess the genetic machinery for the de-novo synthesis of other osmoprotectants viz., glycine betaine and trehalose^22^ and therefore, proline may be used as the major osmoprotectant under acid induced osmotic stress. Many bacteria use glutamate as osmoprotectant under different environmental stresses^19,41–43^. *B. megaterium* can use glutamate to activate another acid tolerance mechanism called glutamate decarboxylase system and therefore, glutamate may not be involved in conferring osmoprotection. The improved growth of *B. megaterium* under acid stress after supplementing the medium with glutamate can be linked to the induction of glutamate decarboxylation system as reported earlier^31^. Moreover, glutamate may also aid for intracellular proline biosynthesis as glutamate acts as a precursor for proline biosynthesis. However, in the *proC* mutant cells, production of proline from glutamate is not possible (as the *proC* gene is inactive) and therefore only the gad system can be functional in acidic pH. As a result of this, the mutant cells showed slower growth rate after glutamate supplementation, compared to the supplementation of extracellular proline.

The spectrophotometric and HPLC analysis revealed that l-proline is produced and secreted or released to the extracellular medium under acid and salt stress in 6 h. Therefore, we aimed to analyze the expression of the *proC* gene at 1 h and 5 h under the same conditions and in the presence of proline (1 mM). The qPCR analysis revealed increased expression of the *proC* gene in WT during its growth in acid stress condition at 1 h irrespective of the presence or absence of proline indicating that at 1 h the cells do not produce sufficient amount of proline required to protect the cells from acid stress. By 5 h, the expression of the *proC* in acid-stressed WT cells in presence and absence of proline did not vary significantly (p ≤ 0.05) compared to the non-stressed WT cells. This indicates that when the intracellular level of proline reaches a certain concentration, its biosynthesis decreases and a part of the intracellular proline may be exported outside the cell as revealed by results of the spectroscopy and HPLC analysis. Although, the fluorescent microscopy study revealed a possibility of leakage of intracellular proline into the media from the dead cells or the cells with leaky membrane (Fig. 7), we think that secreted amount of proline will be very less given that only a small fraction of the bacterial population were dead or had leaky membrane. Our present study revealed that the bacterium released the proline in to the medium only after 5 h of growth under acid stress condition. The extracellular proline production was initially measured using a colorimetric method^44^. To avoid any ambiguity in the experimental method, we designed another experiment where the culture filtrate of *B. megaterium* G18 grown for 16 h (pH 7.0 and 4.5) was used as medium for growing *E. coli* proline auxotrophs. Minimal media (pH 7.0 and 4.5) with and without l-proline (1 mM) supplementation served as positive and negative control, respectively. The survival of the *E. coli* proline auxotrophs in minimal media supernatant and in the l-proline supplemented media indicated that *B. megaterium* G18 secreted proline into the media which helped the *E. coli* auxotrophs to survive whereas, the same mutants could not survive in the medium that had not been supplemented with l-pronine.

Involvement of proline biosynthesis in acid tolerance was further confirmed by expression of full length *proC* gene in *B. megaterium* proC mutant cells. Induced expression of *proC* gene using pHT01 plasmid vector complemented the production of Δ^1^-pyrroline-5-carboxylate reductase, which in turn allowed the proline biosynthesis, thereby supported the growth of mutant cells in acidic pH.

Although *B. megaterium* WT cells experienced osmotic stress in acidic pH condition as indicated by their altered morphology, supplementation of proline in the medium allowed the cells to overcome the osmotic stress and regain their original cell shape. This indicates that acid stress induces osmotic stress in *B. megaterium* which, the bacterium overcomes by either producing proline or taking it up from external source. Our study provides strong evidences of the requirement of proline by the *B. megaterium* cells to survive under acid stress condition (pH 4.5). It will be interesting to see if proline is involved in other possible mechanisms which may be operational in *B. megaterium* cells growing under acid stress. Further studies may shed more light in understanding its role under acid stress condition.

## Methods and materials

### Bacterial strains, growth media, plasmids and oligonucleotides primers

Bacterial strains used in this study include *B. megaterium* G18^30^ and *E. coli* S17.1 with resistance to trimethoprim (Tp^r^) and streptomycin (Sm^r^). The *E. coli* strain also harbored the suicide plasmid pSUP5011^32^ which was used as donor bacteria for Tn5 mutagenesis. The integration vector pMUTIN4 (BGSC, OSU, USA) was used for targeted gene inactivation in *B. megaterium* G18. The bacterial strains and plasmids used in this study are listed in Tables 1 and 2. The pMUTIN4 vector contained the *bla* (β-lactamase) gene that confers ampicillin-resistance (100 µg/ml) required for maintaining the plasmid in *E. coli* and *ermAM* gene that encodes rRNA adenine N-6-methyltransferese (*ermAM*) selectable in Gram-positive bacteria and confers resistance to erythromycin (0.3 µg/ml)^45,46^. *B. megaterium* G18 was grown either in Nutrient broth (NB; Himedia, India) or in minimal medium (MM) (Supplementary file, Table S1), with appropriate antibiotics as and when required. The *E. coli* strain was grown in Luria Broth (LB; Himedia, India) with or without antibiotics as required. Hydrochloric acid (HCl) was used to adjust the pH of the media when required. The media was solidified with agar powder (Difco, USA) at 15 g/L when required. Concentrations of antibiotics for *E. coli* strain S17.1/pSUP5011::Tn5-mob were as follows: nalidaxic acid 50 µg/mL; neomycin, 50 µg/mL and chloramphenicol, 30 µg/mL. For *E. coli* proline auxotrophs, kanamycin (50 µg/mL) was used. For selection of *B. megaterium*, neomycin (50 µg/mL) and erythromycin (0.3 µg/mL) were added to the medium as per requirement. The growth of the organisms was monitored by measuring the optical density at 600 nm in a Spectroquant Pharo 300 spectrophotometer (Merck, Germany). Oligonucleotide primers used in this study are given in Table 3.

**Table 1:** Details of bacterial strains used in this study.

Sl. no.	Strain/isolate	Genotype	Reference
1	*Bacillus megaterium* G18	Wild type	^1^
2	*E. coli* TOP10	F-, *mcrA, *Δ(*mrr-hsd*RMS-*mcr*BC) Φ80*lac*ZΔM15 Δ *lac*X74 *rec*A1 *ara*D139 Δ(*araleu*)7697 *gal*U *gal*K *rps*L (StrR) *end*A1 *nup*G	Invitrogen™, USA
3	*E. coli S17.1*	*pro, res*^*−*^* hsdR17 (rK*^*−*^* mK*^+^*) recA*^*−*^ with an integrated *RP4-2-Tc::Mu-Km::Tn5*, Tp^r^	^2^
4	*E. coli* K12- JW0233-2 (CGSC#: 8468)	F-, *Δ(araD-araB)567*, *ΔproA761::kan*, *ΔlacZ4787*(::rrnB-3), *λ-*, *rph-1*, *Δ(rhaD-rhaB)568*, *hsdR514*	CGSC, USA^a^
5	*E. coli* K12-JW0232-1 (CGSC#: 8467)	F-, *Δ(araD-araB)567*, *ΔproB760::kan***,** *ΔlacZ4787*(::rrnB-3), *λ-*, *rph-1*, *Δ(rhaD-rhaB)568*, *hsdR514*	CGSC, USA
6	*E. coli* K12-JW0377-1 (CGSC#: 8554)	F-, *Δ(araD-araB)567*, *ΔlacZ4787*(::rrnB-3), *ΔproC751::kan*, *λ-*, *rph-1*, *Δ(rhaD-rhaB)568*, *hsdR514*	CGSC, USA
7	Wild type, *E. coli* K12-MG1655, CGSC#: 6300	F-, λ-, rph-1	CGSC, USA
8	*Bacillus megaterium* BA5	Neo^r^	This study
9	*Bacillus megaterium* 13A (ΔproC)	Erm^R^	This study
10	*Bacillus megaterium* ΔproC (pHT01-proC^+^)	Chloramphenicol resistance (Cm^R^), Erm^R^	This study

^a^CGSC: Coli Genetic Stock Center, Yale University, USA.

**Table 2:** Details of plasmids used in this study.

Sl. no.	Vectors	Characteristics	Reference
1.	pSUP:Tn5:mob	pBR325::Tn*5-mob* with ampicillin [Ap^r^], chloramphenicol [Cm^r^], and kanamycin resistance [Km^r^] and a P-type-specific recognition site for mobilization	^2^
2.	pMUTIN4	Integration vector for *Bacillus* spp. containing Ap^r^ and Erm^R^ (erythromycin resistance)	BGSC, USA^a^
3.	pMutProCi	Derivative of pMUTIN4 containing PCR amplified fragment proCi, Ap^r^ and Erm^R^	This study
4.	pHT01	Expression vector for *Bacillus* spp. with Ap^r^ and Cm^r^	MoBiTec, USA
5.	pHT01-proC	Derivative of pMUTIN4 containing PCR amplified full length proC gene, Ap^r^ and Cm^r^	This study

^a^BGSC: Bacillus Genetic Stock Center, Ohio State University, USA.

**Table 3:** Details of oligonucleotides used in this study. The restriction sites within the oligonucleotide sequences are mentioned in italics.

Sl. no.	Primer name	Sequences (5′ → 3′)	Target gene and purpose of use
1.	NptII-F	TAGACTGGGCGGTTTTATGGACAG	*nptII* (encodes neomycin phosphotransferase II enzyme that metabolizes neomycin and kanamycin and gives resistance against these antibiotics to the organism harboring this gene). Used to detect the presence *nptII* gene in the transformants produced after transposon mediated mutagenesis
	NptII-R	AACTCCGCGAGGTCGTCCAGCCTC	
2.	MproC-F	CTTC*AAGCTT*CCAGCTTGTCCTATCTGTTCTT	*proC* (encodes pyrroline-5-carboxylate reductase, one of the genes involved in proline synthesis). Used to amplify the internal fragment of *proC* and its subsequent cloning into pMUTIN4 for targeted inactivation of *proC*
	MproC-R	CGTT*GGATCC*TCAGGATCGAGACCTTCTTCT	
3.	ermAM-F	GAACAAAAATATAAAATATTCTCG	*ermAM* (encodes the enzyme Adenine methylase that metabolize erythromycin and gives resistance against these antibiotics to the organism harboring this gene) Used to detect the presence *ermAM* gene in the transformants produced after transformation of pMutproCi
	ermAM-R	TCCTCCCGTTAAATAATAGATAACT	
4.	RT16S-F	GTGTCGTGAGATGTTGGGTTA	16S rRNA gene (used as endogenous control in qRT-PCR)
	RT16S-R	GTGTGTAGCCCAGGTCATAAG	
5.	RTproC-F	GGAAGTGGACCCGCTTATTT	*proC* (used to measure the differential expression of *proC* through qRT-PCR
	RTproC-R	TCGGCGTTTCATCTCTTTCC	
6.	Exp-proC-F	CC*TCTAGA*ATGGCAGAAGCGATGATTTCTG	*proC* (used to amplify the internal fragment of *proC* gene for cloning into pHT01 plasmid and expression analysis)
	Exp-proC-R	CG*CCCGGG*TTAATTAGTACTTGCCACTTTTTGTAG	

### Transpositional mutagenesis

The Tn*5* mutagenesis was performed through conjugation of *B. megaterium* G18 with the donor (*E. coli* S17.1) as described earlier^47^. *B. megaterium* cells were grown in NB while, the *E. coli* S17-1 carrying pSUP5011 was cultivated in LB broth containing neomycin (50 µg/mL) and incubated overnight at 37 °C. Separate 250-ml Erlenmeyer flasks each containing 50 ml NB and LB medium were subsequently inoculated with 1 ml of overnight *B. megaterium *G18 and *E. coli* S17-1 cultures respectively. The cultures were grown at 37 °C to obtain an OD_600_ of 0.6–0.8. Cells were harvested by centrifugation (15 min, 4000 rpm, 4 °C), washed twice in 15 mL holding buffer (12.5 mM KH_2_PO_4_, 12.5 mM K_2_HPO_4_, 1 mM MgSO_4_, pH 7.2). This step was repeated once and the pellet obtained was resuspended in 30 mL holding buffer. The *E. coli* cells were stored at 4 °C until use. The *B. megaterium* G18 cells were subjected to heat treatment for 9 min at 49 °C prior to the conjugation process where both donor and recipient cells were mixed in the ratio of 1:2 (donor/recipient). The culture mixture was centrifuged at 4000 rpm for 10 min at 4 °C and the pellet resuspended in 0.5 mL holding buffer and laid over a sterile nitrocellulose filter (pore size of 0.45 μm) to ensure close contact between donor and recipient cells. The filter was placed on sporulation agar^48^ with the cells forming the top layer and allowed to incubated for 3 days at 30 °C for sporulation. Since *B. megaterium* produces endospore, counter-selection against *E. coli* S17.1 was done by pasteurization. Cells in the filter were suspended in 900 μL holding buffer and incubated at 80 °C for 20 min. The heat treated cells were then spread on LB agar plates containing neomycin (50 µg/mL). To determine the conjugation efficiencies, cells were spread in dilutions (10^−5^ to 10^−7^) on LB agar plates and incubated for 24 h at 37 °C. Conjugation efficiencies were calculated as the number of trans-conjugants divided by the whole number of *Bacillus* spores determined as colony forming units after the heat treatment (80 °C).

### Pheno- and geno-typic analyses of the Tn5 mutants

The trans-conjugants were tested for their acid tolerance/susceptibility by growing them in NB adjusted to pH 4.5. Trans-conjugants that could not survive at pH 4.5 were regarded as acid susceptible and selected for further study. A genotypic analysis of the knockout mutants was performed by PCR using primer set NptII-F and NptII-R (Table 3). The amplification of was performed as per GoTaq^®^ DNA polymerase protocol (Promega, USA) in a final volume of 50 μL with 20 pmol of each primer, 2U Taq DNA polymerase and approximately 50 ng of *B. megaterium* genomic DNA using a thermal cycler (Applied Biosystems, USA). The PCR program was as follows: initial denaturing step of 94 °C for 3 min, followed by 35 cycles of denaturation at 94 °C for 30 s, annealing at 60 °C for 1 min and extension at 72 °C for 1 min 30 s; a final extension step at 72 °C for 7 min^49^.

### Isolation of Tn5 mutant requiring proline for growth at low pH and growth curve analysis

The acid susceptible mutants were checked for the requirement of l-proline during their growth at pH 4.5. The mutant strains were allowed to grow in NB and minimal media of pH 7.0 and 4.5 with and without l-proline and the growth of the cells were monitored for 12 h and compared with the growth of the wild type. The growth of the cells at both conditions was monitored by plating the serially diluted culture on NA plate. The colonies obtained were counted and represented as logCFU/mL.

### Targeted disruption of *pro*C gene

The targeted disruption of *proC* gene was performed using the integration vector pMUTIN4^45^, obtained from Bacillus Genetic Stock Center (BSGC, USA). This is a systemic gene inactivation vector for *Bacillus* sp. which specifically, contains the origin of replication (*ori*) that functions in *E. coli* but not in Gram positive bacteria. When an internal fragment of the target open reading frame (ORF) is cloned into the cloning site of this vector and transformed into appropriate host cell, the plasmid integrates into the host chromosome through Campbell type recombination. This result in disruption of the targeted gene and the integrant(s) can be selected on nutrient agar plates containing 0.3 µg/ml of erythromycin^45,46^.

The genomic DNA of *B. megaterium* G18 was isolated using NucleoSpin Microbial DNA kit (Macherey–Nagel, Germany) as described by the manufacturer. To amplify an internal fragment of *proC* gene designated as *proC*i, the nucleotide sequence of *proC* gene of *B. megaterium* ATCC14581 was retrieved from Gene database (www.ncbi.nlm.nih.gov/gene) and primers (Table 2) were designed from the retrieved sequence using Primer Quest tool (https://eu.idtdna.com/PrimerQuest) such that the forward primer contains HindIII restriction site and the reverse primer contains the BamHI site. The amplification of *proC*i was performed as per GoTaq^®^ DNA polymerase protocol (Promega, USA) in a final volume of 50 μL with 20 pmol of each primer, 2U Taq DNA polymerase and 50 ng of *B. megaterium* G18 genomic DNA using a thermal cycler (Applied Biosystems, USA). The PCR program was as follows: initial denaturing step of 94 °C for 3 min, followed by 35 cycles of denaturation at 94 °C for 30 s, annealing at 60 °C for 1 min and elongation at 72 °C for 30 s; a final extension step at 72 °C for 7 min. The amplified *proC*i along with pMUTIN4 were then digested with HindIII and BamHI. The digested products were purified using NucleoSpin^®^ Gel and PCR Clean-up kit (Macherey–Nagel, Germany) and the purified *proC*i fragment was ligated to digested pMUTIN4 using T4 DNA ligase (Takara, Japan) to produce the recombinant plasmid pMUTproCi. The ligated products were transformed into chemically competent *E. coli* TOP10 cells (Invitrogen™ USA). The recombinant plasmid pMUTproCi (Supplementary file, Fig. S1) was isolated using Wizard® Plus SV Minipreps DNA Purification System kit (Promega, USA) and used to transform *B. megaterium* G18 protoplasts as described previously^50^.

### Phenotypic analysis of the *proC* mutants

The colonies that appeared in the NA plates with erythromycin were sub-cultured for three consecutive generations. The colonies that showed resistance to antibiotic in the plates were considered as mutants and tested for their cultural characteristics such as acid susceptibility and growth pattern. The mutants and the WT were grown freshly in NB till OD_600_ of 1.0. The cultures (6 logCFU/mL) were then used to inoculate NB and minimal media of different pH (7.0 and 4.5) and incubated at 37 °C with shaking (150 rpm) for 12 h. The growth of the cells in both the conditions was monitored by plating the serially diluted culture on NA plate. The colonies that appeared on the plates were counted and represented as logCFU/mL. The mutant cells were also tested for their ability to grow in saline condition by growing them in NB and minimal media (pH 7.0) supplemented with 500 mM and 1 M NaCl.

### Confirmation of mutants through PCR amplification of *ermAM* gene

The acid susceptible isolates were confirmed for the insertion of pMUTproCi into the chromosome by PCR amplification of *ermAM* gene using the genomic DNA of the mutants as template. The DNA of the mutants was isolated by NucleoSpin Microbial DNA isolation kit (Macherey–Nagel, Germany). The primer set ermAM-F and ermAM-R (Table 2) was designed using Primer Quest software form the nucleotide sequences of the pMUTIN2 vector (GeneBank accession number: AF072806.1), the parent vector of pMUTIN4 to amplify *ermAM* gene. The *ermAM* gene was then amplified using the primer set ermAM-F and ermAM-R in a thermal cycler (2720 Thermal cycler, Applied Biosystem^®^, USA) as per GoTaq^®^ DNA polymerase protocol (Promega, USA) in a final volume of 50 μL with 20 pmol of each primer, 2U Taq DNA polymerase and 50 ng of genomic DNA. The PCR program was as follows: initial denaturing step of 94 °C for 3 min, followed by 35 cycles of denaturation at 94 °C for 30 s, annealing at 60 °C for 1 min and elongation at 72 °C for 45 s; a final extension step at 72 °C for 7 min. The amplified PCR products were resolved in 1% agarose gel and visualized under UV. The presence of ~ 700 bp single band indicates the amplification of *ermAM* gene. The plasmid pNUTIN4 and genomic DNA of *B. megaterium* G18 was used as positive and negative control respectively. Among the acid susceptible *ermAM* positive isolates, the isolate 13A was considered for further analysis.

### Effect of different osmoprotectants on the growth of *B. megaterium* WT and mutant (13A) in acid stress condition

Studies have shown that bacteria produce proline mostly under osmotic stress^22,38,39^. Therefore, we hypothesized that the acid susceptible phenotype of the *proC* mutant was due to the acid stress induced osmotic imbalance. In order to test our hypothesis, the mutant 13A and the WT were grown under acid stress condition (pH 4.5) in presence of 3 different osmoprotectants viz*.*, glutamate, beataine, and l-proline. Briefly, both wild type and the mutant were grown in minimal media of pH 7.0 till OD_600_ of 1.0. One milliliter (6 logCFU/mL) of wild type and mutant cultures was then transferred to minimal media (pH 4.5) with and without adding 1 mM each of glutamate, beataine, and l-proline in separate conical flask and allowed to grow for 12 h. Growth was monitored every 2 h by plating the serially diluted culture on NA plate. The colonies obtained were counted and represented as logCFU/mL.

### Measurement of extracellular proline concentration

The amount of extracellular proline secreted by the bacteria was determined by growing the wild type cultures at 37 °C for 1 h and 6 h in 50 mL minimal medium adjusted to pH 7.0 and 4.5 and minimal media (pH 7.0) containing 500 mM NaCl under shaking (150 rpm) conditions. The cultures were harvested by centrifugation at 10,000 rpm at 4 °C for 10 min and the supernatant was used for measuring the proline concentration as described previously^44^ with some modifications. A 2-mL of the culture supernatant was mixed with 1200 μL of sulphosalicylic acid (3% aqueous solution) and centrifuged at 13,000 rpm for 10 min at 4 °C. After centrifugation, 500 μL of supernatant was transferred into a clean test tube and the volume was made 1 mL using sterile H_2_O. This step was followed by the addition of 1 mL of glacial acetic acid and 1 mL of ninhydrin (2% solution prepared in acetone). The prepared samples were incubated for 1 h in a water bath at 90 °C. The samples were cooled on an ice bath, which was followed by the addition of toluene (2 mL) and mixing for 2 min using a vortex mixture. The supernatant was collected in a fresh tube and the absorbance at 520 nm was measured on a spectrophotometer^44^. Proline concentrations were estimated using the standard curve of proline prepared from serial dilutions of proline stock solutions and expressed as mM of proline in the supernatant.

### Validation of extracellular proline production using *E. coli* proline auxotrophs

To validate the production of extracellular proline by *B. megaterium* G18 under acid stress, *E. coli* proline auxotrophs (Table 2) were inoculated in filtered culture supernatant of *B. megaterium* G18. The culture filtrate was obtained by inoculating *B. megaterium* cells in MM (50 mL) at pH—7.0 and 4.5 (adjusted with HCl) for 6 h under shaking (150 rpm) at 37 °C. Thereafter, the cells were harvested by centrifugation (10,000 rpm, 10 min) and the supernatant obtained was filtered through 0.2 µm filter. Glucose was added to the filtered supernatant at the concentration of 10 g/L. The *E. coli* proline auxotrophs were incubated at 37 °C in a shaker incubator (150 rpm) and the growth was monitored at an interval of every 1 h till 5 h.

### Measurement of extracellular proline production using HPLC

An HPLC analysis was performed to measure the amount of proline produced in the extracellular medium. The *B. megaterium* cells were grown in minimal media of pH 7.0 and 4.5 for 1 h, 6 h and 16 h. l-proline being recognized as an osmolyte associated with osmotic stress, a control experiment was set up by inoculating the cells in minimal media (pH 7.0) supplemented with 500 mM NaCl. The cells were harvested after the required time of incubation and the cell free filtered supernatant was used for the HPLC analysis after derivatization. Derivatization was performed using diethyl ethoxymethylene melanoate (DEMM) (Sigma, USA) as described earlier^51^. The mobile phase consisted of phase A: 25 mM sodium acetate (pH 5.8) with 0.02% sodium azide; phase B: 100% acetonitrile. Twenty microlitre of the sample was injected into a Cosmosil C-18 column (Nacalai Tesque, Inc., Japan) through the autosampler of Hitachi Chromaster 5000 series HPLC system (Hitachi, Japan) and peaks were detected at 280 nm using a diode array detector. The elution was performed with a flow rate of 1.0 ml/min as follows: 6% B at 0–2.5 min, 14% B at 6–9 min, 20% B at 11 min, 26% B at 13.5–15 min, 50% B at 17–18.5 min, 63% B at 20–21.5 min, 100%B at 23–55 min and equilibrating the column with 6% B at 26–30 min. Standard solutions of proline at different concentrations were prepared and derivatization was performed in the same way and a standard curve was prepared from the peak area of each standard.

### qRT-PCR analysis of *proC* gene

The spectrophotometric and HPLC analysis revealed that l-proline is produced and secreted to the extracellular medium under acid and salt stress of 6 h. Therefore, we aimed to check the expression of the *proC* gene at 1 h and 5 h. For qRT-PCR analysis the cells were grown in minimal media as described for HPLC analysis. The cells were harvested after 1 h and 5 h incubation and total RNA were extracted using PureLink RNA Mini kit as per the given protocol (Thermo Scientific, USA). The total RNA was then converted to first strand cDNA using Goscript cDNA synthesis kit (Promega, Madison, USA). Quantitative real-time PCR was performed on the first strand cDNA using GoTaq qPCR Master Mix (Promega, Madison, USA) in a total reaction volume of 20 μl containing 10 pmol primers and 50 ng cDNA template according to the manufacturer’s instructions. Real-time PCR was performed using three biological replicates on the QuantStudio 5 real-time PCR System (Applied Biosystems, USA). The 16S rRNA gene was used as the reference gene. The relative gene expression data were analyzed using the 2^−ΔΔCt^ method^52^. The primers used for the qRT-PCR analysis is shown in Table 1.

### Expression of full length *proC* gene in acid susceptible ∆proC mutant

The full length *proC* gene from *B. megaterium* genomic DNA was amplified using the forward and reverse primers (Exp-proC-F and Exp-proC-R) containing the restriction sites for XbaI and XmaI, respectively at the 5′ ends (Table 3). For expression of the gene, pHT01 vector system (MoBiTec, Germany) containing those two restriction sites was used. Restriction digestion was performed using XbaI and XmaI (New England Biolabs, USA) restriction enzymes following the manufacturer’s instructions. Purification of the restriction digested products was performed using Nucleospin Gel and PCR Cleanup kit (Takara, Japan) as per the manufacturer’s instructions. Ligation of the restriction digested vector and insert was performed, which was subsequently cloned into *E. coli* TOP10 cells as described in the previous section (see “Targeted disruption of *proC* gene”). Transformed colonies containing the pHT01-proC plasmid were selected on LB agar plates containing ampicillin (50 mg/L) and screened for the presence of full length proC gene using the same primers. Plasmid DNA isolated from the positive clones was then transformed into *B. megaterium* ΔproC protoplasts (as described previously^50^) and the transformed colonies were selected on nutrient agar plates containing 5 mg/L chloramphenicol and 1 mM IPTG.

### Confirmation of *proC* gene expression in ∆proC mutant cells

Transformation of pHT01-proC plasmid into the *B. megaterium* ΔproC cells was confirmed through colony PCR based amplification of the *bla* gene using the primers mentioned in Table 2). The plasmid DNA was recovered from the transformed cells using Nucleospin plasmid purification kit (Macherey–Nagel, Germany), and PCR amplification of the *proC* gene was confirmed using gene specific primers. For expression analysis, the RNA content was extracted from *B. megaterium* wild type cells, *B. megaterium* ΔproC cells and *B. megaterium* ΔproC cells harbouring pHT01-proC plasmid (designated as *B. megaterium* pHT01-proC^+^). The cells were exposed to pH 4.5 for 1 h and 5 h prior to RNA extraction. RNA and 1st strand cDNA synthesis were performed as described in the previous section (see “qRT-PCR analysis of *proC* gene”). Expression of the proC gene was checked using quantitative real-time PCR, where the amplification of 16S RNA gene was used for normalization. *Bacillus megaterium* wild type cells exposed to pH 7.0 for similar duration were used as reference for qPCR analysis.

### Growth analysis of *B. megaterium* pHT01-proC^+^ cells

To study the growth characteristics of *B. megaterium* pHT01-proC^+^ cells was analyzed in minimal medium (pH 4.5 and pH 7.0) as described in the previous section (see “Effect of different osmoprotectants on the growth of *B. megaterium* WT and mutant (13A) in acid stress condition”). The growth curve was prepared by taking the OD_600 nm_ value at respective time points and compared to that of *B. megaterium* wild type and *B. megaterium* ΔproC cells grown at pH 7.0 and pH 4.5. A 1 mM proline supplementation was used to compensate the proline production in *B. megaterium* ΔproC.

### Fluorescence microscopy

In order to check whether acid stress caused death of some cells or produced cells with leaky membrane a fluorescent microscopic analysis was performed. Bacterial cells were grown in in minimal media of pH 7.0 and 4.5 as well as in minimal media (pH 7.0) supplemented with 500 mM NaCl and allowed to grow the cells for 6 h and 16 h. One milliliter of the cells from each culture was centrifuged and washed twice in 1 ml of 0.22 μm filtered PBS then stained using the Live/Dead^®^ BacLight™ bacterial viability kit (Invitrogen, USA) following the manufacturer’s protocol. Briefly, the bacterial cells were incubated in the dark at room temperature for 5 min with 0.5 μL each of 1.67 mM Propidium iodide (PI) (red dye) and 1.67 mM SYTO9 (green dye). The microscopic observations were performed using an Olympus BX 51 Fluorescence Microscope (Olympus Corporation, Japan).

### Bright-field microscopy

To observe for any morphological changes that might occur during acid induced osmotic stress, we performed a basic microscopic analysis. The WT and 13A were grown in minimal media of pH 7.0 till OD_600_ of 1.0 was achieved. Then one milliliter of wild type and mutant cultures were transferred to fresh minimal media (pH 4.5) with and without adding 1 mM each of glutamate and l-proline in separate conical flask and allowed to grow for 1 h. In addition, both WT and 13A cells were also allowed to grow in MM containing 500 mM NaCl at pH 7.0 with and without the addition of l-proline for 1 h. The cells were then harvested and observed under microscope after Gram staining and the cell shape of WT and 13A cells grown in different conditions were compared.

### Statistical analysis

At least three independent replicates of each experiment were performed if not mentioned otherwise. The results of real time PCR were statistically analyzed using one way analysis of variance (ANOVA) in SPSS (ver. 25.0). Duncan multiple range test was performed to study the level of significance among the treatments (p ≤ 0.05).

## Conclusion

Although, proline is known to play several important functions in bacteria including providing protection against biotic and abiotic factors, its function in conferring acid tolerance in bacteria has hardly been reported. In order to understand the importance of proline in providing protective mechanism against low pH condition, an acid susceptible mutant of *B. megaterium* requiring proline for its growth was generated and selected. Proline requirement by the mutant was confirmed by its inability to grow in pH 4.5 in absence of l-proline. To further understand, the mechanism of protective mechanism of proline under acidic condition, the *proC* gene coding for Δ^1^_-_pyrrolione-5-carboxylate reductase was disrupted in *B. megaterium* through pMUT1N4. The mutant was unable to grow in acidic condition (pH4.5) in absence of l-proline and had distorted cellular morphology. Exogenous addition of proline revived the growth of the mutant cells in acidic condition and helped alleviate osmotic induced stress on the cells. Further, plasmid based expression analysis of the full length *proC* gene in the *proC* mutant cells could restore survival capacity of the mutants in acidic pH, suggesting its active role in acid tolerance to *B. megaterium* G18. Although, our study has shown for the first time the importance of proline in conferring acid tolerance in *B. megaterium* G18; detailed studies on other facet of the involvement of proline will aid in gaining better understanding of its role in acid tolerance in bacteria.

## Supplementary Information

Supplementary Information.

## Data Availability

Data generated in this study are provided as supplementary information to this manuscript. Bacterial strains will be available upon conditional request to the corresponding author. The authors declare that the work presented in the manuscript has not been published previously nor it is under consideration for publication elsewhere.
