# Supplementary material for: Proline confers acid stress tolerance to Bacillus megaterium G18
Source: Sci Rep. 2022 May 25;12:8875. doi: 10.1038/s41598-022-12709-0 (PMC9133035; doi:10.1038/s41598-022-12709-0)
Supplement: Supplementary file 1 — Supplementary Information. [file 41598_2022_12709_MOESM1_ESM.pdf]

# Proline confers acid stress tolerance to *Bacillus megaterium* G18

Table S1: Composition of minimal media used to grow *B. megaterium* G18

| *Ingredients | Quantity (gm/L) |
|---|---|
| Di-potassium phosphate ($K_2HPO_4$) | 7.000 |
| Sodium citrate ($Na_3C_6H_5O_7$) | 0.500 |
| Magnesium sulphate ($MgSO_4.7H_2O$) | 0.500 |
| Ammonium chloride ($NH_4Cl$) | 1.000 |
| Ammonium nitrate ($NH_4NO_3$) | 1.000 |
| Calcium chloride ($CaCl_2$) | 0.050 |
| Ferric chloride ($FeCl_3$) | 0.003 |
| Final pH ( at 25°C) | 7.0±0.2 |

*The above constituents were suspended in 1000 ml distilled water and stirred under heat until completely dissolved. It was sterilized by autoclaving at 15 lbs pressure (121°C) for 15 minutes, cooled to Room temperature and then 10 ml of 10% filter sterile Dextrose solution was added

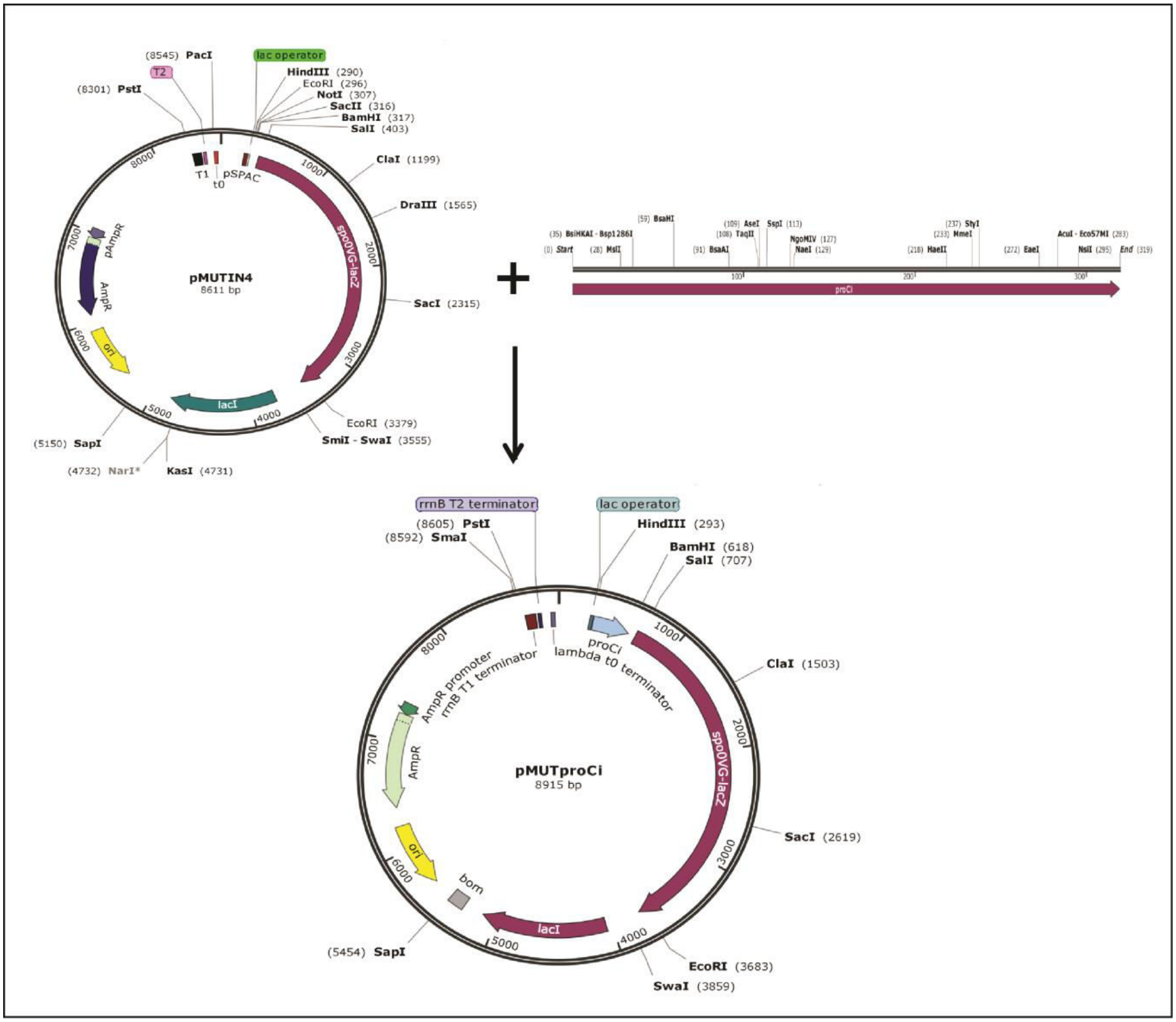


Fig S1 Construction of pMUTproCi. The integration vector pMUTIN4 and an internal fragment of *proC* (proCi) were digested in parallel with BamHI and HindIII. The digested vector and the PCR fragment were ligated using T4 DNA ligase to obtain the pMUTproCi

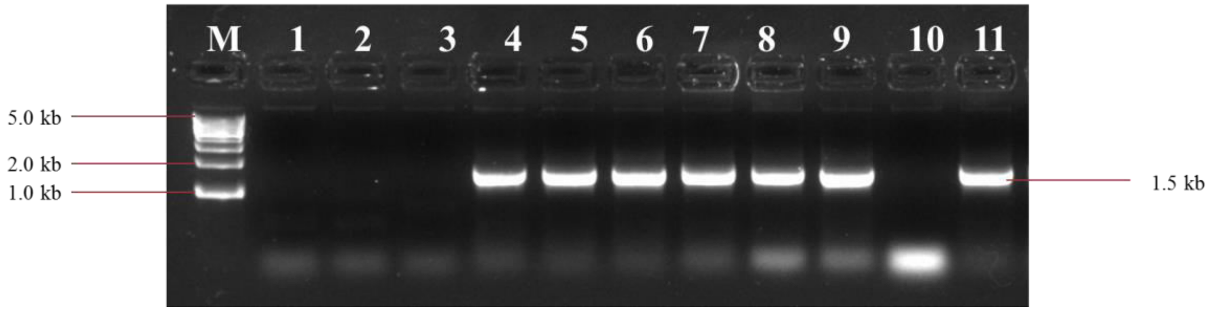


Fig S2 Confirmation of transposon mutants of *B. megaterium* G18 through PCR amplification of *nptII* gene. Lane-M: 1.0 kb Molecular ladder (Takara); Lane-1-9: Transconjugants; Lane-10-Wild type; Lane-11: Positive control (pSUP5011)

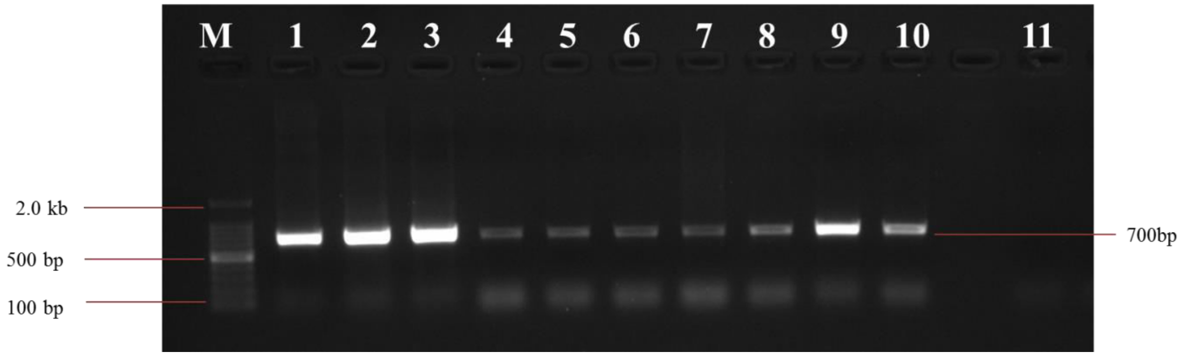


Fig S3 Validation of integration of pMUTproCi into the chromosome of *B. megaterium* G18 through PCR amplification of *ermAM* gene. M-100 bp DNA ladder (Takara), 1: Positive Control (pMUTIN4); 2-10: mutant; 11- WT

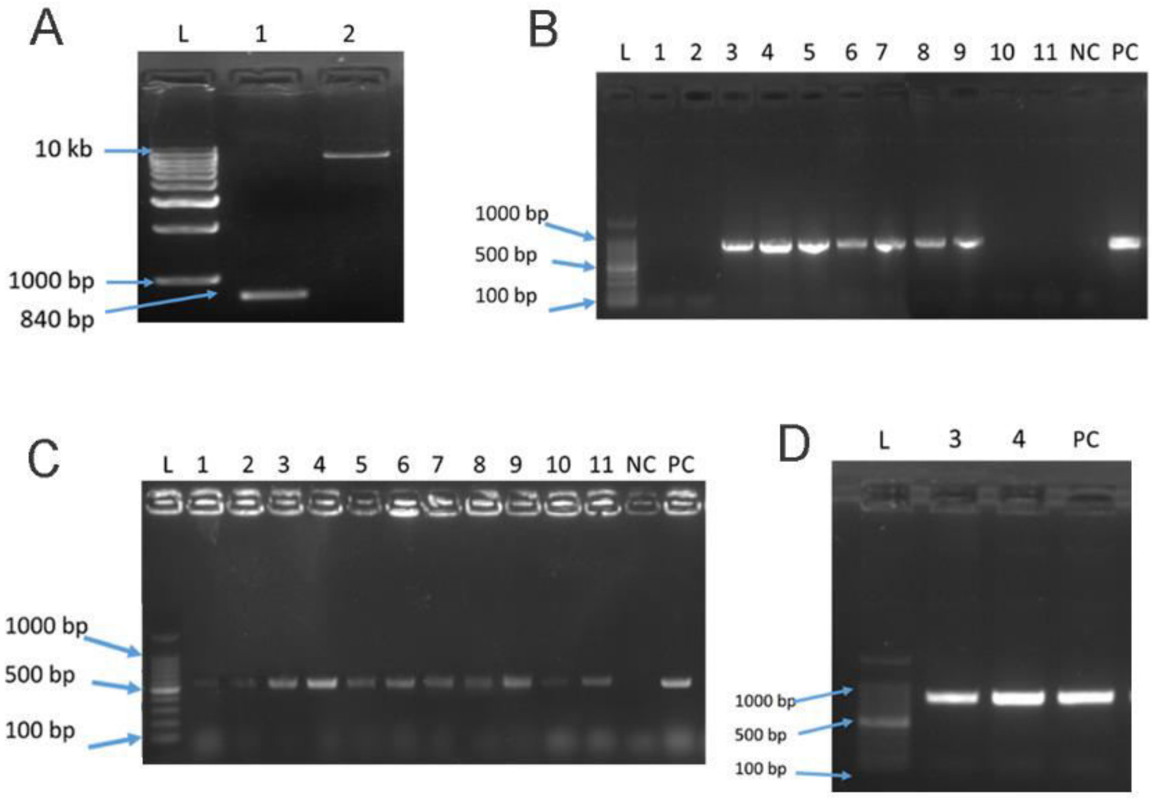


Fig. S4: Expression of full length proC gene in *B. megaterium* ΔproC cells using pHT01 expression vector. A. Restriction digested products of (1) full length proC gene containing flanking restriction sites for XbaI and XmaI, and (2) pHT01 vector; B. Colony PCR based amplification of the full length proC gene in the *E. coli* Top10 colonies transformed with the ligated pHT01-proC construct; C. Colony PCR amplification of *bla* gene to confirm the presence of pHT01-proC plasmid inside *B. megaterium* ΔproC cells after transformation; D. Amplification of bla gene from the plasmids isolated from *B. megaterium* ΔproC (pHT01-proC+) cells (from colony 3 and 4 of Fig. C). NC denotes negative control, where no colony/DNA template was added, while PC denotes positive control containing genomic DNA from B. megaterium G18 (for Fig. B), or pHT01 plasmid containing *bla* gene.

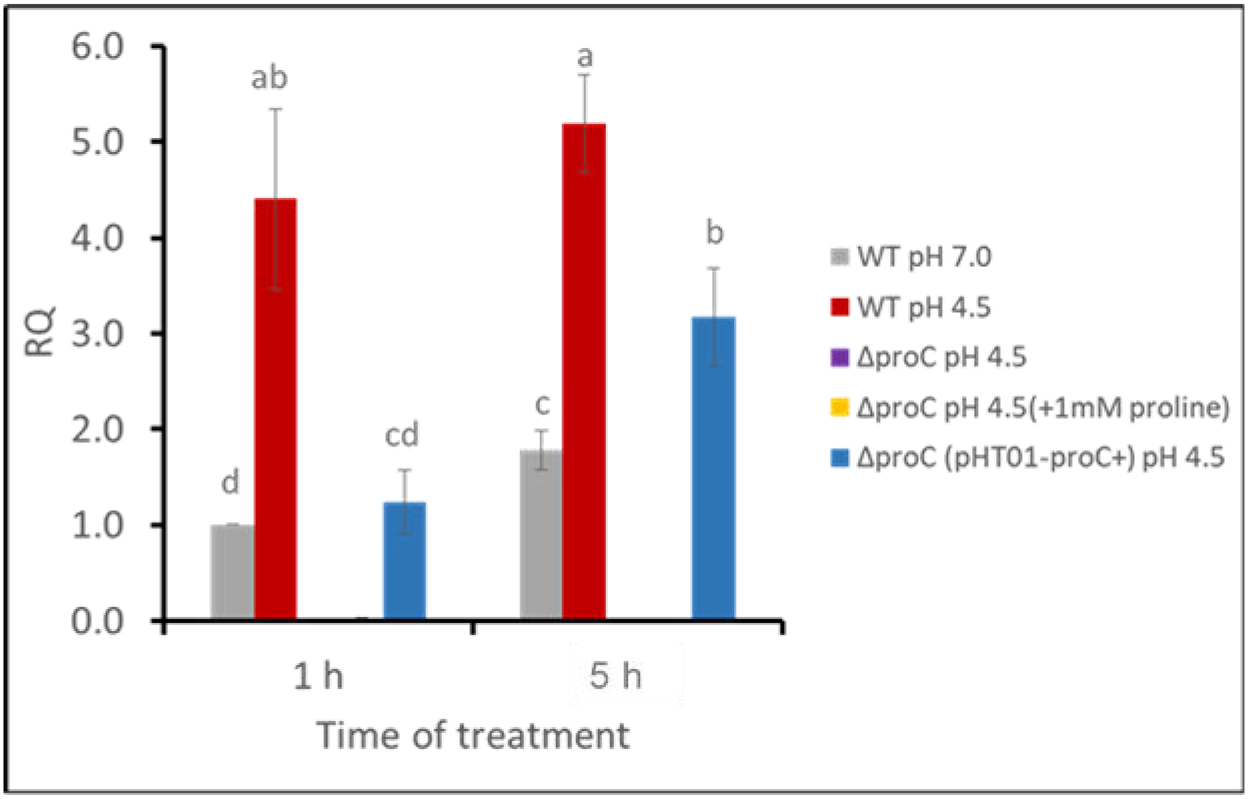


Fig. S5: Quantitative real-time PCR based expression analysis of the proC gene in wild-type, proC mutant and pHT01-proC containing proC mutant cells after 1 h and 5 h of incubation at respective conditions. No expression was observed in the proC mutant cells (even after addition of 1 mM proline) as the gene was inactivated. Different letters above the bars indicates the level of significance calculated using one way ANOVA and Duncun multiple range test ($p \leq 0.05$)

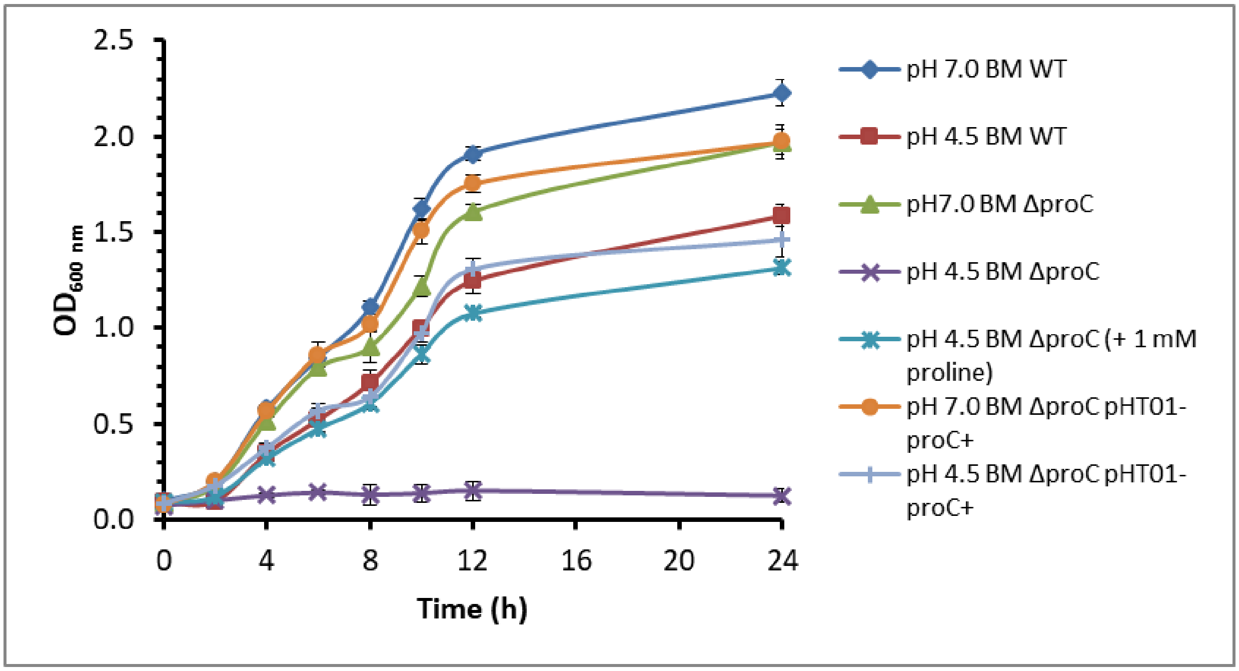


Fig. S6: Bacterial growth curve of *B. megaterium* wild type and mutant cells at different conditions.
